# PD-1 activation mitigates lupus nephritis by suppressing hyperactive and heterogeneous PD-1^+^CD8^+^ T cells

**DOI:** 10.7150/thno.107418

**Published:** 2025-03-31

**Authors:** Jun Deng, Junling Zhu, Xiaoyue Jiang, Chao Yao, Haifeng Chen, Yanjie Ding, Peng Niu, Qian Chen, Huihua Ding, Nan Shen

**Affiliations:** 1 Shanghai Institute of Rheumatology, Department of Rheumatology, Renji Hospital, Shanghai Jiao Tong University School of Medicine, Shanghai, China.; 2 State Key Laboratory of Systems Medicine for Cancer, Shanghai Cancer Institute, Renji Hospital, Shanghai Jiao Tong University School of Medicine, Shanghai, China.; 3 Department of Rheumatology and Immunology, Huaihe Hospital of Henan University, Kaifeng, 475000, Henan, China.; 4 Department of Ophthalmology, Tongren Hospital, Shanghai Jiao Tong University School of Medicine, Shanghai, China.; 5 Center for Autoimmune Genomics and Etiology (CAGE), Cincinnati Children's Hospital Medical Center, Cincinnati, OH, United States.

**Keywords:** Lupus nephritis, PD-1+CD8+ T cell, scRNA-seq, TCR clonality

## Abstract

**Rationale:** Programmed cell death protein 1 (PD-1)-expressing CD8^+^ T cells are typically associated with exhaustion in cancer and infections, but their role in autoimmune diseases, particularly lupus nephritis (LN), remains less understood. Understanding the characteristics and functions of PD-1^+^CD8^+^ T cells in LN could help identify novel therapeutic strategies.

**Methods:** We analyzed the abundance and phenotypes of PD-1^+^CD8^+^ T cells in LN patients and NZB/W F1 mice. Single-cell RNA sequencing (scRNA-seq) was used to delineate subsets and TCR clonal diversity in PD-1^+^CD8^+^ T cells in NZB/W F1 mice. The therapeutic efficacy of a PD-L1 Fc fusion protein on kidney pathology and proteinuria in NZB/W F1 mice was evaluated. In addition, the inhibitory mechanism of PD-1 in CD8^+^ T cells were further explored using RNA-seq, q-PCR, flow cytometry, and Western blot.

**Results:** PD-1^+^CD8^+^ T cells were enriched in LN patients and NZB/W F1 mice, exhibiting elevated activation markers and cytotoxic molecules compared to PD-1^-^ cells. scRNA-seq identified seven distinct subsets with diverse effector functions and robust TCR clonal expansion in the kidney of NZB/W F1 mice with severe disease. PD-L1 Fc treatment reduced kidney damage and proteinuria in NZB/W F1 mice, which correlated with decreased frequencies of PD-1^+^CD8^+^ and IFN-γ^+^CD8^+^ T cells. Mechanistically, PD-L1 Fc inhibited Stat1 phosphorylation, T-bet expression, and IFN-γ production in CD8^+^ T cells.

**Conclusion:** These findings show that PD-1^+^CD8^+^ T cells in LN are hyperactive, clonally expanded, and contribute to disease progression. Targeting the PD-1/PD-L1 pathway with PD-L1 Fc effectively reduced kidney pathology in a murine model of LN, underscoring the potential of modulating PD-1 signaling as a treatment strategy for LN.

## Introduction

Systemic lupus erythematosus (SLE) is a chronic autoimmune disease characterized by systemic inflammation and immune dysregulation 1, 2. Lupus nephritis (LN), a major complication, significantly contributes to SLE-related morbidity by involving autoantibody production and proinflammatory mediator release 3-5. CD8^+^ T cells, while typically protective in chronic infections and cancer 6, can also drive tissue damage and worsen autoimmunity 7-9, as demonstrated by their infiltration into the kidney and association with disease activity in LN 10. These cells manifest cytotoxic, memory effector, and tissue-resident phenotypes in LN patients 11-13. However, certain CD8^+^ regulatory T cell subsets have protective roles in LN by reducing autoreactive B cells and promoting immune regulation 14, 15, adding complexity to the overall picture. This dual role of CD8^+^ T cells in LN highlights their contributions to both pathology and immune homeostasis.

The programmed cell death-1 (PD-1)/PD-L1 axis is crucial in maintaining immune balance 16. Genetic variations in *PDCD1* influence LN susceptibility, with certain polymorphisms providing protective effects 17, 18. In murine models, PD-1 deficiency fosters proliferative glomerulonephritis 19. While anti-PD-1/PD-L1 therapies have transformed cancer treatment, they also lead to immune-related adverse events (irAEs), including lupus erythematosus (LE) onset 20, 21. Among reported cases of LE as irAEs, 93.2% were linked to anti-PD-1/PD-L1 21, underscoring the complex role of PD-1 in LN.

Although CD8^+^ T cell exhaustion is well-known in cancer and chronic infections 6, 22, its role in LN remains unclear. Initial reports suggest that exhaustion markers might correlate with better outcomes in SLE patients 23, and studies in MRL/*lpr* mice indicate that kidney-infiltrating CD8^+^ T cells are metabolically and functionally exhausted 24. However, more recent single cell RNA-sequencing (scRNA-seq) data reveal diverse CD8^+^ T cell populations in LN, including cytotoxic and tissue-resident memory phenotypes, without hallmark signs of exhaustion 25, 26. These conflicting findings suggest a more nuanced role of "exhausted" CD8^+^ T cells in LN that requires further exploration.

In this study, we demonstrate that PD-1^+^CD8^+^ T cells are enriched in the kidney of both LN patients and a murine model, exhibiting a hyperactivated phenotype and promoting disease progression. These populations consist of diverse subsets, featuring clonally expanded effector and cytotoxic populations in NZB/W F1 mice. Notably, PD-L1 Fc treatment significantly ameliorated disease in NZB/W F1 mice by inhibiting PD-1^+^CD8^+^ T cells and reducing IFN-γ production via the Stat1-T-bet axis. These findings highlight that hyperactivated PD-1^+^CD8^+^ T cells play a pivotal role in LN and indicate that targeting the PD-1 pathway as a promising therapeutic strategy.

## Methods

### Human samples

Twenty patients fulfilling the 1997 American College of Rheumatology classification criteria for LN 27 were recruited. Blood samples and renal biopsies were collected with written informed consent and approved by the Ethical Committee of Renji Hospital, Shanghai Jiao Tong University School of Medicine (KY-2022-03-B). Detailed patient demographics are provided in Supplementary tables 1-2 (Table S1 and Table S2).

### Mice and PD-L1 Fc treatment

Female NZB/W F1 mice were obtained from Jackson Laboratory (Bar Harbor, USA), and C57BL/6 mice from Shanghai Jihui Laboratory Animal Care Co., Ltd. (Shanghai, China). All animal experiments were conducted in accordance with the Institutional Animal Care and Use Committee (IACUC) guidelines of Renji Hospital, Shanghai Jiao Tong University School of Medicine. Beginning at 20 weeks of age, female NZB/W F1 mice received intraperitoneal injections of recombinant mouse PD-L1/B7-H1 Fc chimera protein (100 µg/mouse, R&D Systems) or PBS twice weekly through 24 weeks of age. Mice were then analyzed at 25 weeks.

### Cell culture

CD8^+^ T cells were isolated from female NZB/W F1 mice and cultured in 96-well plates coated with anti-CD3 (1 µg/mL, BioLegend) and anti-CD28 (1 µg/mL, BioLegend) antibodies, with or without PD-L1 Fc (0-5 µg/mL, R&D Systems), for 72 h. For proliferation assays, CD8^+^ T cells were labeled with carboxyfluorescein succinimidyl ester (CFSE, 1 µg/mL) and stimulated with anti-CD3/CD28 antibodies in the presence or absence of PD-L1 Fc for 72 h.

### Isolation of kidney-infiltrating immune cells

Renal biopsies from LN patients or renal tissues from NZB/W F1 mice were minced and digested with collagenase I (1 mg/mL) at 37°C for 10 min. The resulting cell suspension was layered on a 40% Percoll gradient and centrifuged at 500 g for 20 min at 4°C. The mononuclear cell (MNC) layer between 40% and 80% Percoll was collected and washed with PBS containing 5% FBS and 1% penicillin/streptomycin.

### Flow cytometry

Single-cell suspensions of human and mice samples were stained with fluorochrome-conjugated antibodies. For mouse samples, the following antibodies were used: CD4 (RM4-5), CD8a (53-6.7), CD44 (IM7), CD62L (MEL-14), IFN-γ (XMG1.2), TNF-α (MP6-XT22), granzyme B (QA16A02), perforin (eBioOMAK-D), Foxp3 (FJK-16s), CD38 (90), CD69 (H1.2F3), CD103 (2E7), OX40 (OX-86), CD29 (HMβ1-1), T-bet (4B10), Ki-67 (B56), and active caspase 3 (C92-605). For human samples, antibodies included TCRαβ (IP26), CD19 (SJ25C1), CD4 (SK3), CD8 (SK1), CD45RA (HI100), CD62L (DREG-56), TNF-α (MAb11), IFN-γ (B27), IL-21 (3A3-N2) and perforin (dG9). These antibodies were obtained from BD Biosciences, BioLegend, or ThermoFisher. For intracellular staining, cells were stimulated for 5 h with PMA (Sigma-Aldrich), ionomycin (Sigma-Aldrich), monensin (BioLegend), or brefeldin A (BD Pharmingen). Intracellular antigens were stained using the Fixation/Permeabilization Solution Kit (BD Pharmingen). Zombie Aqua™ Fixable Viability Kit (BioLegend) was used to distinguish live/dead cells. Data were acquired on a flow cytometer (BD Fortessa X20) and analyzed with FlowJo software (BD).

### ELISA

Serum anti-dsDNA IgG and ANA levels from NZB/W F1 mice were measured using the ELISA kit (Alpha Diagnostic International) in accordance with the manufacturer's instructions. Serum IFN-γ from NZB/W F1 mice with IgG or PD-L1 Fc treatment, and IFN-γ in cell culture supernatant of CD8^+^ T cells were determined by the ELISA MAX™ Standard Set Mouse IFN-γ kit (BioLegend) following the manufacturer's protocols.

### Bulk RNA-sequencing, single-cell RNA and V(D)J sequencing

Splenic PD-1^+^CD44^+^CD8^+^ and PD-1^-^CD44^+^CD8^+^ cells were sorted, and total RNA was extracted using the RNeasy Mini Kit (QIAGEN). RNA quality was verified on an Agilent 2100 Bioanalyzer. Libraries were prepared with SMART-seq and sequenced on the Illumina HiSeq X Ten platform.

For scRNA-seq, PD-1^+^CD44^+^CD8^+^ cells were sorted and processed using the Chromium Controller (10× Genomics). Libraries were prepared with the Chromium Single Cell 5′ v2 Reagent Kit and quality-checked using a Qubit fluorometric assay and Agilent 2100 Bioanalyzer. Sequencing was performed on the HiSeq2500 platform, yielding 100 million reads per sample and 10,000 reads per cell.

V(D)J sequencing was performed using the Single-Cell V(D)J Enrichment Kit (10× Genomics) to enrich TCR segments from amplified cDNA. The Cell Ranger vdj pipeline (v.3.0.2) was used for demultiplexing, gene quantification, and TCR clonotype assignment using the GRCm38 mouse genome. TCR diversity was assessed with ACE and Chao indices, focusing on productive TCR (TRA) and TCR (TRB) chains. Clonotypes were defined by unique TRA-TRB pairs, and dominant TCR clonotypes were visualized using UMAP. TCR libraries were pooled and sequenced to a depth of 5,000 read pairs per sample.

### Quantitative real-time PCR

Total mRNA from cultured CD8^+^ T cells was extracted using TRIzol reagent (Invitrogen). Complementary DNA (cDNA) was synthesized using the cDNA Synthesis Kit (Takara), and real-time PCR was performed on a QuantStudio™ 7 Flex Real-Time PCR System (Thermo Fisher) with SYBR Green Master Mix (Invitrogen). qPCR primers used for the analysis are listed in Table S3. The relative expression levels of target genes were normalized to *Actb* expression, and data were analyzed using the ΔCt method.

### Western blot

CD8^+^ T cells from NZB/W F1 mice were activated with anti-CD3/CD28 and treated with or without PD-L1 Fc (0-5 μg/mL). Cells were lysed in RIPA buffer (Sigma-Aldrich) containing PMSF (ThermoFisher) and a protease inhibitor cocktail (Roche Diagnostics). Proteins were separated via SDS-PAGE and transferred onto membranes. Primary antibodies included rabbit anti-phospho-Stat1 (Tyr701, 58D6), Stat1 (D1K9Y), and β-Actin (D6A8) from Cell Signaling Technology, as well as anti-T-bet (4B10) from BioLegend. HRP-conjugated anti-rabbit IgG secondary antibodies were used, and protein bands were visualized using chemiluminescent substrates (GE Healthcare) on a ChemiDoc XRS system (Bio-Rad). Band intensity was quantified using ImageJ software (NIH).

### Immunohistochemistry and immunofluorescence staining

Human renal biopsies and NZB/W F1 mouse kidney sections were stained with anti-CD8 antibodies (D8A8Y for human, C8/144B for mouse; CST) and counterstained with Mayer's hematoxylin (Dako). Images were captured using a NanoZoomer S360 Digital Slide Scanner (Hamamatsu).

For human kidney-infiltrating PD-1^+^CD8^+^ T cells, sections were stained with FITC-CD8α (RPA-T8, CST), PD-1 (D7D5W, CST), and Cy3-conjugated donkey anti-rabbit IgG. Mouse kidney sections were stained with goat anti-mouse IgG (Sigma-Aldrich) and Cy3-conjugated donkey anti-goat IgG (Proteintech). PD-1^+^CD8^+^ T cells in mouse kidneys were detected using anti-CD8 (53-6.7, BioLegend) and anti-PD-1 (D7D5W, CST), with nuclei counterstained by DAPI. Confocal images were acquired using a Zeiss LSM 710 microscope and analyzed with Photoshop CC 2019.

### Statistics

Data were analyzed using GraphPad Prism (version 8.0). Comparisons between PD-1^+^CD8^+^ and PD-1^-^CD8^+^ T cells in LN patients and NZB/W F1 mice, as well as between treatment groups (PD-L1 Fc vs. control) in NZB/W F1 mice, were performed using the Mann-Whitney U test. For CFSE-labeled proliferation assays and analysis of IFN-γ and T-bet expression in cultured CD8^+^ T cells, one-way ANOVA was employed. Data are presented as mean ± SEM (or median for human data) and considered statistically significant at p < 0.05.

## Results

### Hyperactivation of PD-1^+^CD8^+^ T cells in LN patients

**Figure 1:**
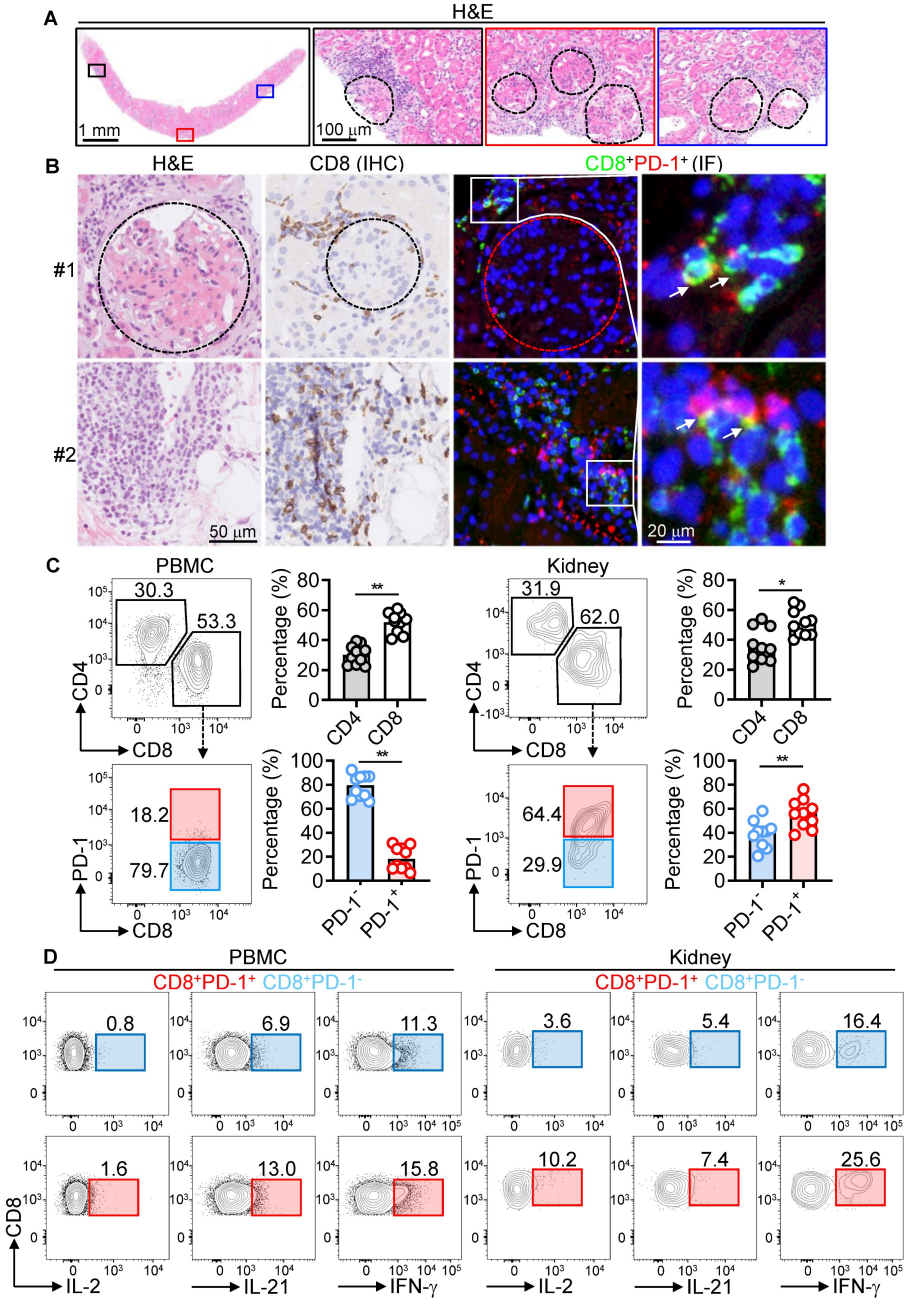
Hyperactivation of kidney-infiltrating PD-1^+^CD8^+^ T cells in LN patients. (A) H&E staining showing immune infiltration and glomerulopathy in renal biopsies from LN patients. The glomerulus is delineated by a dashed box. (B) IHC and IF staining of CD8^+^ and PD-1^+^CD8^+^ T cells in renal biopsies. (C, D) FACS analysis of CD4^+^, CD8^+^, PD-1 expression (C), and IL-2, IL-21, IFN-γ expression in PD-1^+^CD8^+^ and PD-1^-^CD8^+^ T cells (D) from PBMCs and MNCs of renal biopsies (n = 10) stimulated with PMA, ionomycin, and brefeldin A for 5 h. Data are shown as median with interquartile range and analyzed by Mann-Whitney U-test (C). *p ≤ 0.05, **p ≤ 0.01.

Ample studies have highlighted the complexity of CD8^+^ T cells and PD-1 in the pathogenesis of LN 18, 28. To investigate further, we characterized PD-1^+^CD8^+^ T cells in peripheral blood mononuclear cells (PBMCs), and mononuclear cells (MNCs) from renal biopsies of LN patients. Histopathology revealed significant glomerular damage, interstitial lesions, cast formation, and immune cell infiltration (Figure 1A). CD8^+^ T cells were present in these regions, forming large immune aggregates near the glomerular capsule. PD-1^+^CD8^+^ T cells were localized along Bowman's capsule and associated with immune aggregates (Figure 1B).

To elucidate the roles of CD4^+^ and CD8^+^ T cells, we quantified these subsets in PBMCs and renal biopsies using flow cytometry. The CD8^+^/CD4^+^ ratio was higher in both PBMCs (1.8) and renal biopsies (1.9), indicating preferential CD8^+^ T cell infiltration in the kidney. Renal CD8^+^ T cells showed significantly higher PD-1 expression (64.4%) compared to circulating CD8^+^ T cells (18.2%), suggesting that the kidney microenvironment promotes PD-1 upregulation (Figure 1C).

Functional analysis demonstrated that PD-1^+^CD8^+^ T cells from both blood and renal tissue were hyperactivated, producing elevated levels of proinflammatory cytokines (IL-2, IL-21, IFN-γ). Additionally, PD-1⁺CD8⁺ T cells in PBMCs showed higher expression of cytotoxic molecules (CD107a, FasL, GZMB, and perforin) compared to their PD-1⁻ counterparts. However, this increased expression was not observed in kidney MNCs (Figure 1D and Figure S1). These findings indicate a pathogenic role for hyperactivated PD-1^+^CD8^+^ T cells in LN.

### Hyperactivation of PD-1^+^CD8^+^ T cells in female NZB/W F1 mice

**Figure 2:**
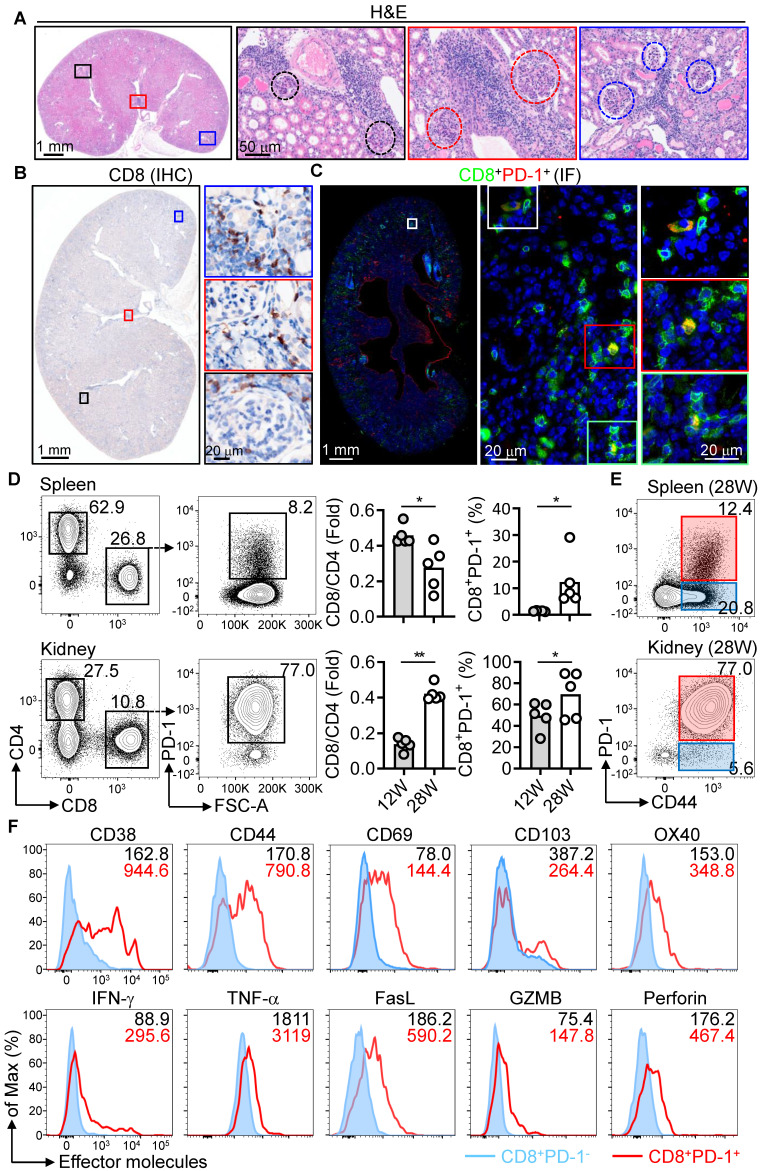
Enhanced activation of PD-1^+^CD8^+^ T cells in NZB/W F1 mice. (A) H&E staining of kidney sections from 28-week-old (28W) female NZB/W F1 mice. The glomerulus is delineated by a dashed box. (B, C) IHC of CD8^+^ T cells (B) and IF of PD-1^+^CD8^+^ T cells (C) in kidney sections from 28W mice. (D-F) FACS analysis of CD4^+^, CD8^+^, PD-1 expression (D), CD44 expression (E), and activation markers/effector molecules on CD8^+^PD-1^+^ and CD8^+^PD-1^-^ T cells (F) from spleen and kidney of 12W and 28W mice. Data are shown as individual points with mean ± SEM and analyzed by Mann-Whitney U-test (D). *p ≤ 0.05, **p ≤ 0.01.

We examined PD-1^+^CD8^+^ T cells in NZB/W F1 mice, a classical murine lupus model 29, at various stages of disease progression. At advanced disease (28 weeks), kidneys exhibited severe damage, including glomerular and interstitial inflammation, fibrosis, and vasculitis (Figure 2A). CD8^+^ T cells accumulated around glomeruli, mesangium, capillary walls, and renal tubules (Figure 2B). PD-1^+^CD8^+^ T cells were observed scattered near glomeruli and renal tubules (Figure 2C).

To further explore T cell dynamics, we quantified CD4^+^ and CD8^+^ subsets in the spleen and kidney at pre-disease (12 weeks) and advanced disease (28 weeks). The CD8^+^/CD4^+^ ratio increased significantly in the kidneys at advanced disease (Figure 2D), correlating with elevated PD-1 expression on CD8^+^ T cells in both spleen and kidney. Most PD-1^+^CD8^+^ T cells in the kidney co-expressed the activation marker CD44 (Figure 2E and Figure S2A). We next analyzed whether PD-1^+^CD8^+^ T cells also express other activation markers. At the pre-disease stage, splenic PD-1^+^CD44^+^CD8^+^ T cells had elevated expression of activation markers (CD38, CD69, OX40), proinflammatory cytokines (IFN-γ, TNF-α), and cytotoxic molecules (FasL, GZMB, perforin) compared to PD-1^-^ counterparts. During advanced disease, kidney-infiltrating PD-1^+^CD44^+^CD8^+^ T cells exhibited significantly higher expression of these markers than PD-1^-^ cells (Figure 2F, Figure S2B-D, and Figure S3). These findings suggest that PD-1^+^CD8^+^ T cells transition from systemic activation at early disease stage to a localized pathogenic phenotype in the kidney during advanced LN.

### Functional heterogeneity and dynamic shifts of PD-1^+^CD8^+^ T cells in NZB/W F1 mice

**Figure 3:**
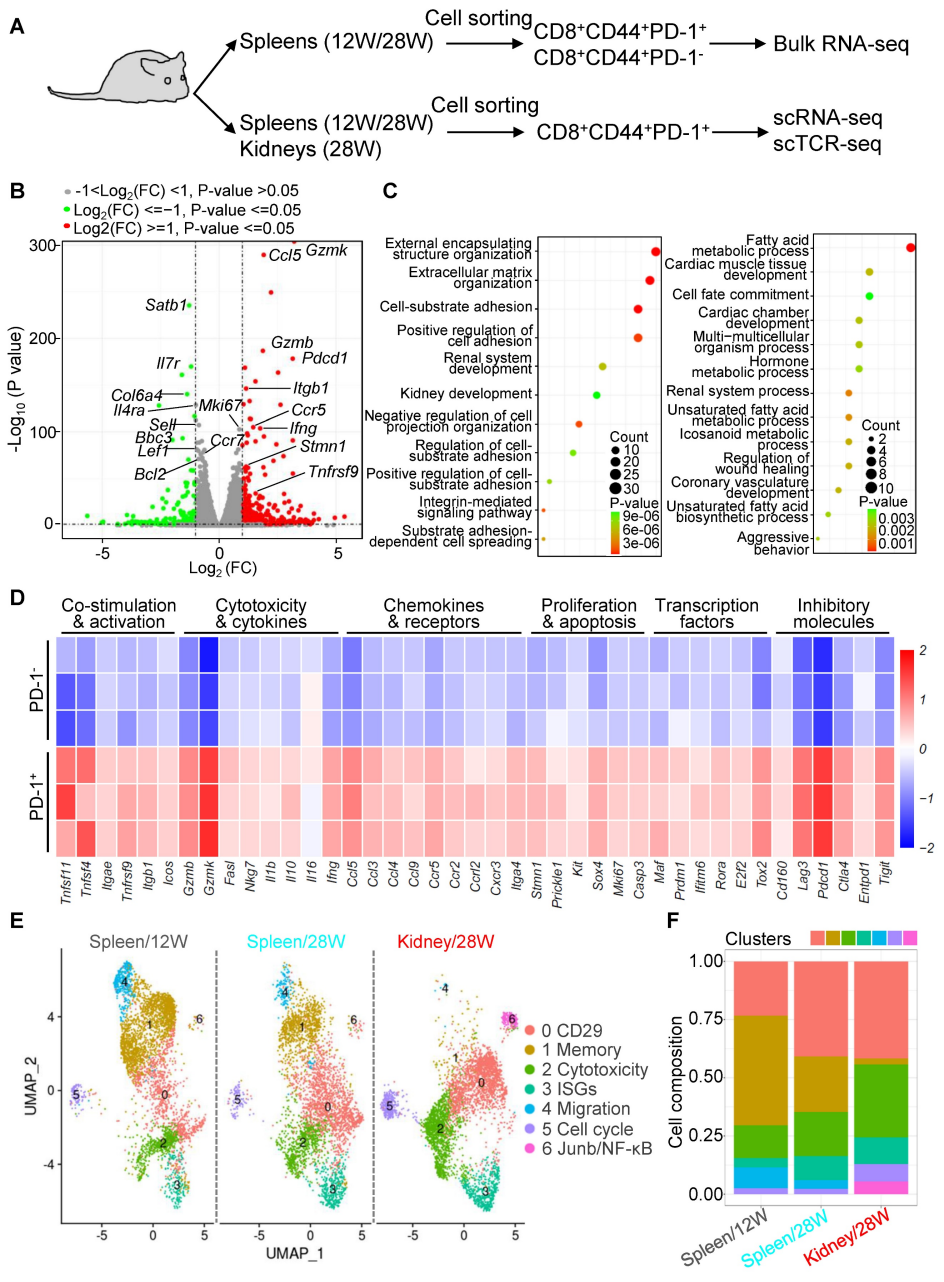
Functional heterogeneity and dynamic shifts of PD-1^+^CD8^+^ T cells in murine LN. (A) Experimental design for transcriptomic analysis of CD8^+^CD44^+^PD-1^+^ and CD8^+^CD44^+^PD-1^-^ T cells from 20-week-old (20W) NZB/W F1 mice. (B-D) Bulk RNA-seq analysis of splenic CD8^+^CD44^+^PD-1^+^ and CD8^+^CD44^+^PD-1^-^ T cells from 20W mice, including a volcano plot for differential gene expression (B), KEGG pathway analysis (C), and heatmap of selected genes (D). (E, F) scRNA-seq of PD-1^+^CD8^+^ T cells from spleens (12W and 28W) and kidneys (28W), showing UMAP plots for cell clustering (E) and cluster distribution (F).

Given the hyperactivation of PD-1^+^CD8^+^ T cells in LN patients and NZB/W F1 mice, we compared PD-1^+^CD44^+^CD8^+^ and PD-1^-^CD44^+^CD8^+^ T cells at pre-disease (12 weeks, 12W) and advanced LN (28 weeks, 28W) stages (Figure 3A). Transcriptomic analysis showed that PD-1^+^CD8^+^ T cells were enriched in genes related to cytotoxicity, migration, and proliferation, whereas PD-1^-^ cells were associated with memory and stem-like phenotypes (Figure 3B). KEGG pathway analysis revealed differences in metabolism and cell migration (Figure 3C), consist with previous report that PD-1 signaling regulates the metabolism of memory CD8^+^ T cells 30. PD-1^+^CD8^+^ T cells expressed higher levels of activation (*Tnfsf11*, *Icos*), cytotoxicity (*Gzmb*, *Ifng*), migration (*Ccl5*, *Cxcr3*), proliferation (*Stmn1*, *Mki67*), transcription factors (*Maf*, *Tox2*), and inhibitory molecules (*Lag3*, *Tigit*) compared to PD-1^-^ cells (Figure 3D).

To further characterize PD-1^+^CD44^+^CD8^+^ T cells, we performed scRNA-seq on these cells from the spleen and kidney at 12W and 28W. Seven distinct clusters were identified: CD29, memory, cytotoxic, interferon-stimulated genes (ISGs), migration, proliferation, and inflammatory signaling (Junb/NFκB) (Figure 3E). However, no previously reported regulatory or exhausted subsets were detected 23, 28. The memory cluster, predominant in the spleen at 12W, diminished at 28W and was absent in the kidney at 28W. In contrast, CD29 and cytotoxic clusters expanded in both spleen and kidney at 28W, mirroring the notion that CD29^+^CD8^+^ T cells are IFN-γ-producing and cytotoxic 31. The migration cluster declined as the disease progressed, while ISGs, proliferation, and Junb/NFκB clusters slightly expanded in the kidney at 28W (Figure 3F). These results highlight the dynamic shift of PD-1^+^CD8^+^ T cells from memory-dominant to cytotoxic phenotypes during LN progression.

### Functional profiles and TCR repertoire of PD-1^+^CD8^+^ T cells in NZB/W F1 mice

**Figure 4:**
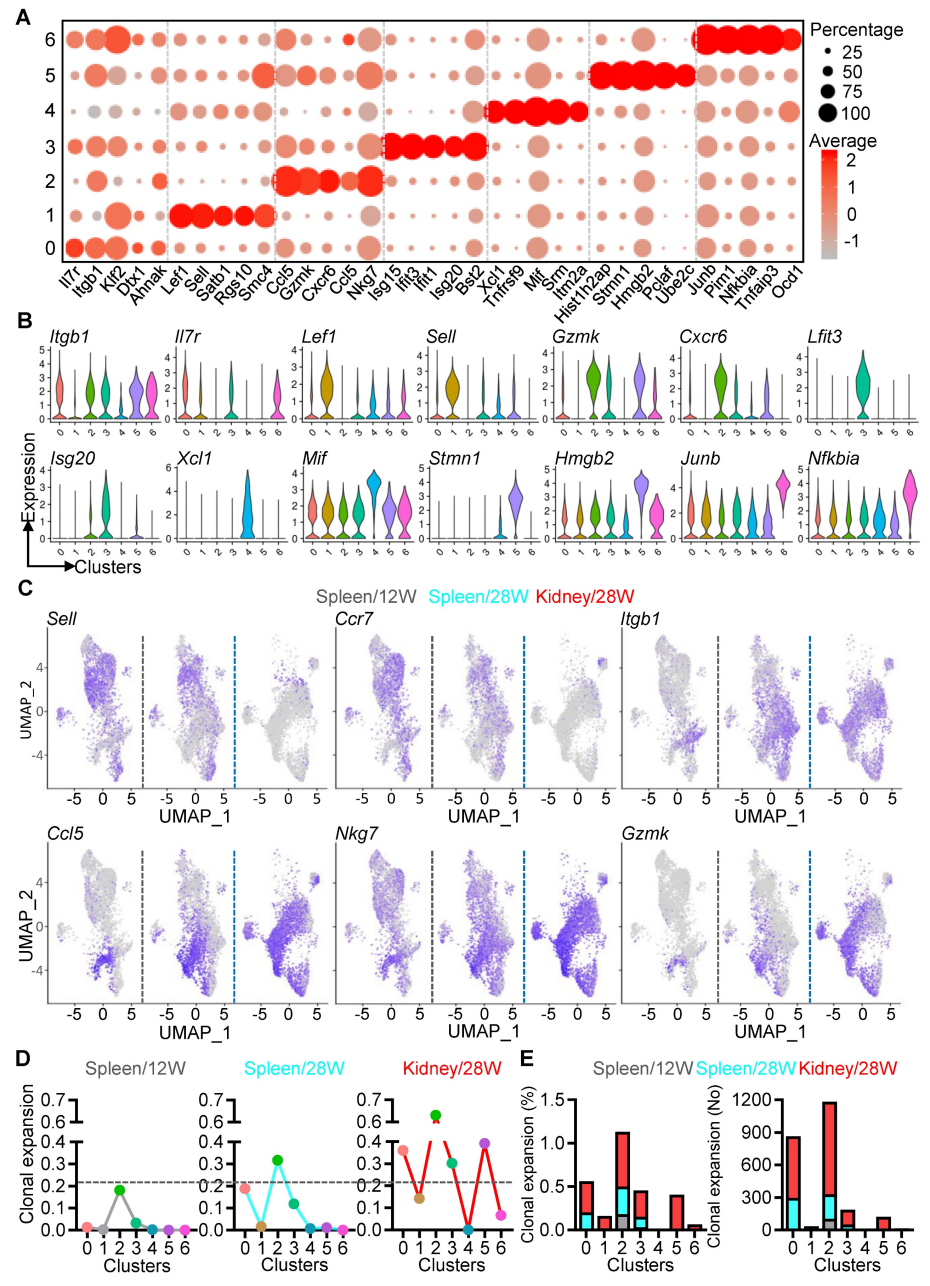
Hyperactivation and robust TCR clonal expansion of PD-1^+^CD8^+^ T cells. (A-C) Gene expression patterns in PD-1^+^CD8^+^ T cell clusters identified by scRNA-seq, visualized using dot plots (A), violin plots (B), and UMAP plots (C) for selected signature genes. (D, E) scTCR-seq analysis of PD-1^+^CD8^+^ T cells from spleens (12W, 28W) and kidney (28W), showing clonal expansion dynamics across clusters (D) and quantification of expanded cells (E).

We conducted an in-depth analysis of gene expression in PD-1^+^CD8^+^ T cell subsets throughout LN progression. Key signature genes were identified for CD29 (*Itgb1*, *Il7r*, *Klf2*), memory (*Lef1*, *Sell*, *Satb1*), cytotoxicity (*Gzmk*, *Cxcr6*, *Nkg7*), interferon response (*Ifit3*, *Ifit15*, *Isg20*), migration (*Xcl1*, *Mif*, *Srm*), proliferation (*Stmn1*, *Hmgb2*, *Pclaf*), and inflammatory signaling (*Junb*, *Nfkbia*, *Tnfaip3*) (Figure 4A). Notably, dynamic shifts in gene expression were observed, with central memory markers (*Sell*, *Ccr7*) prevalent at pre-disease, whereas genes linked to tissue infiltration (*Itgb1*, *Ccl5*) and cytotoxicity (*Nkg7*, *Gzmk*) becoming dominant during advanced disease (Figure 4B-C).

As the clonal expansion of CD8^+^ T cells drive the progression of autoimmune disease 32, 33, we next assessed T cell clonality using single-cell TCR sequencing (scTCR-seq). Limited clonal expansion was observed in most clusters, except for cytotoxic clusters in the 12W spleen. At 28 weeks, the CD29 and cytotoxic clusters exhibited significant expansion in the spleen, while all clusters except migration showed increased expansion in the kidney compared to the spleen (Figure 4D). The cytotoxic cluster had the highest clonal expansion (0.63), followed by proliferation (0.4), CD29 (0.35), ISGs (0.3), and memory (0.14) in the 28W kidney. Moreover, clonally expanded cells were more abundant in the 28W spleen compared to 12W, with robust expansion in CD29, memory, cytotoxic, and ISGs clusters in the 28W kidney (Figure 4E). These findings illustrate the dynamic clonal expansion of PD-1^+^CD8^+^ T cells, shifting towards cytotoxic and effector phenotypes as LN progresses.

### PD-L1 Fc treatment mitigates lupus nephritis in NZB/W F1 mice

**Figure 5:**
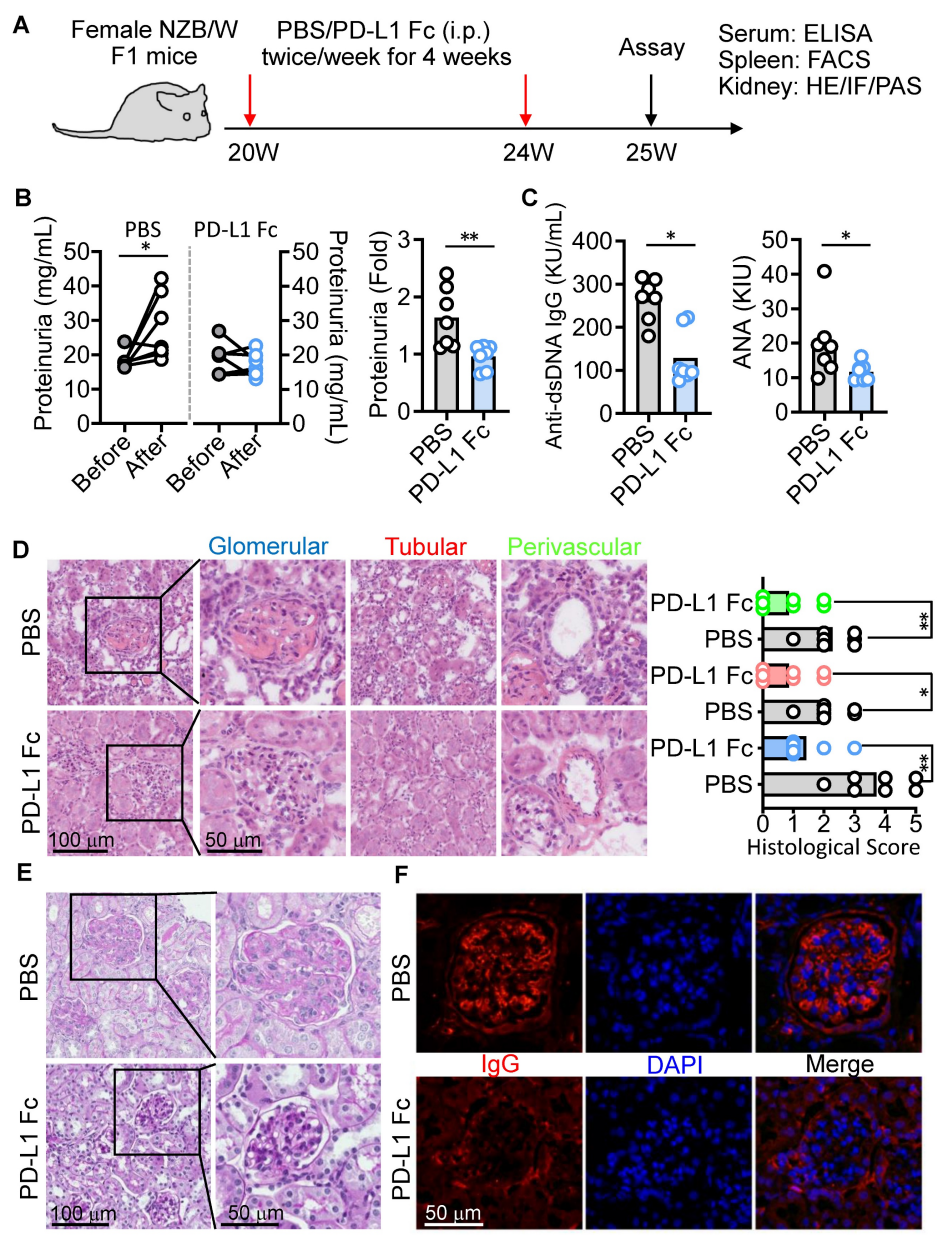
PD-L1 Fc treatment ameliorates kidney damage in NZB/W F1 mice. (A) Experimental design for treatment regimen and outcome measures in female NZB/W F1 mice. (B, C) Proteinuria levels (B) and serum anti-dsDNA and ANA titers (C) in PBS- and PD-L1 Fc-treated mice. (D-F) Kidney histopathology (H&E staining) and scoring (D), PAS staining (E), and IF staining for IgG deposits (F) in PBS- and PD-L1 Fc-treated mice. Data are shown as individual points with mean ± SEM and analyzed by Mann-Whitney U-test (b-d). *p ≤ 0.05, **p ≤ 0.01.

Given that anti-PD-1 therapy expands PD-1^+^CD8^+^ T cells in cancer 34 and can paradoxically induce lupus-like autoimmunity 21, we hypothesized that activating the PD-1 pathway could mitigate LN. To test this, we administered PD-L1 Fc fusion protein to female NZB/W F1 mice between 20 and 24 weeks of age (Figure 5A). PD-L1 Fc treatment significantly alleviated kidney damage compared to PBS-treated controls, notably reducing proteinuria--a key marker of kidney dysfunction (Figure 5B). Serum levels of anti-dsDNA and ANA antibodies were also markedly lower in PD-L1 Fc-treated mice (Figure 5C). Furthermore, significant improvements in kidney pathology, including reduced glomerular, tubular, and perivascular damage scores, were observed (Figure 5D). Mesangial matrix expansion and IgG deposition within glomeruli were substantially diminished, indicating reduced inflammation and tissue damage (Figure 5E-F). These results demonstrate that PD-L1 Fc treatment effectively ameliorates kidney damage in this lupus model.

### PD-L1 Fc suppresses PD-1^+^CD8^+^ T cells and IFN-γ production in NZB/W F1 mice

**Figure 6:**
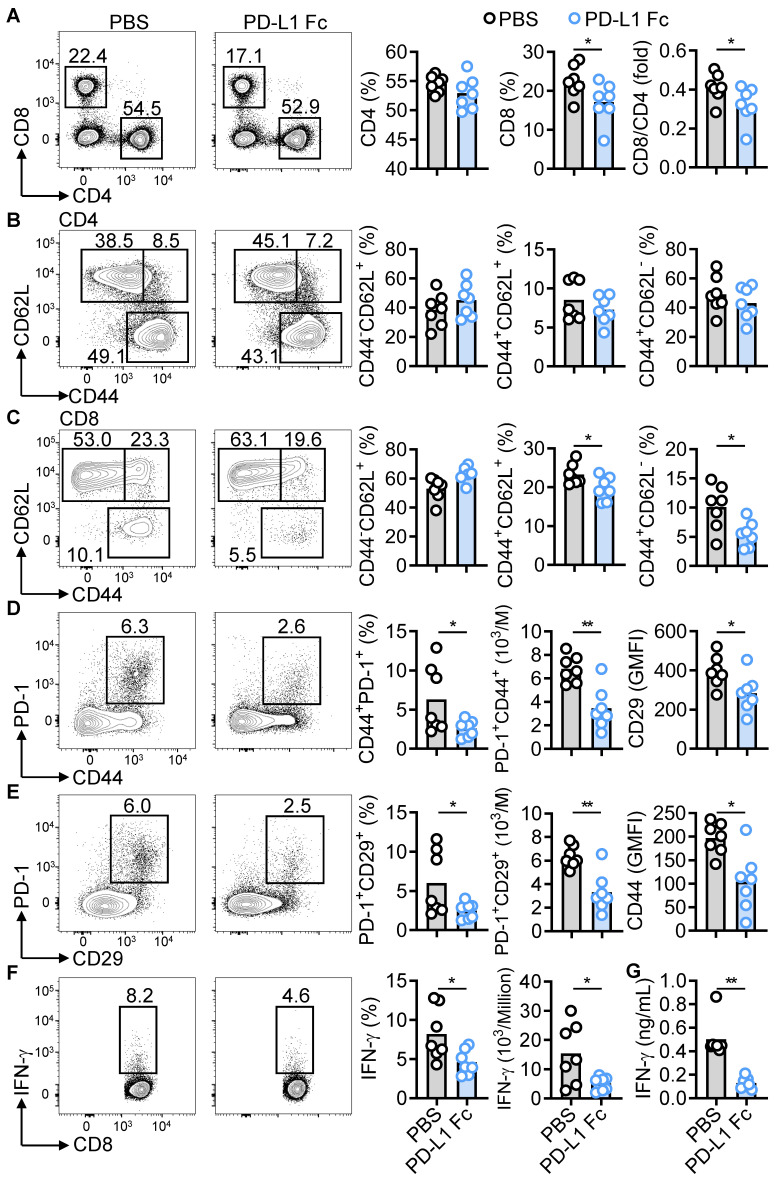
PD-L1 Fc inhibits PD-1^+^CD8^+^ T cell subsets and IFN-γ production. (A-F) FACS analysis of CD4^+^ and CD8^+^ T cells (a), CD4^+^ T subsets (B), CD8^+^ T subsets (C), CD44^+^PD-1^+^CD8^+^ T cells (D), PD-1^+^CD29^+^CD8^+^ T cells (E), and IFN-γ-producing CD8^+^ T cells (F) in spleens of PBS- and PD-L1 Fc-treated mice (n = 7). (G) Serum IFN-γ levels in PBS- and PD-L1 Fc-treated mice. Data are shown as individual points with mean ± SEM and analyzed by Mann-Whitney U-test (A-G). *p ≤ 0.05, **p ≤ 0.01.

Previous studies indicate that targeting CD4^+^ and CD8^+^ T cells with a DNA methyltransferase inhibitor can improve lupus pathology 35. In PD-L1 Fc-treated NZB/W F1 mice, the proportion of splenic CD4^+^ T cells remained unchanged, but the proportion of CD8^+^ T cells and the CD8^+^/CD4^+^ ratio significantly decreased (Figure 6A). PD-L1 Fc treatment had no effect on CD4^+^ T-cell subsets (naïve, memory, effector), but significantly downregulated IFN-γ-producing Th1 cells and upregulated regulatory T cells (Tregs) (Figure 6B and Figure S4A-B), supporting previous findings that the PD-1/PD-L1 axis converts Th1 cells into Tregs 36. T follicular helper (Tfh) and T follicular regulatory (Tfr) cells play critical roles in lupus pathogenesis 37. PD-L1 Fc treatment reduced the proportion of CD4⁺CD44⁺CXCR5⁺PD-1⁺ Tfh cells, while the CD4⁺Foxp3⁺CXCR5⁺PD-1⁺ Tfr cell population remained largely unaffected. This treatment resulted in a minor increase in the Tfr/Tfh ratio, although this change was not statistically significant (Figure S4C-D). In contrast, Th17 cells and TNF-α-producing CD4⁺ T cells were unaffected (Figure S4E-F), indicating selective regulation by PD-1 signaling.

In CD8^+^ T cells, both memory and effector subsets were reduced following PD-L1 Fc treatment (Figure 6C). Pathogenic subsets, including CD44^+^CD29^+^, CD44^+^PD-1^+^, PD-1^+^CD29^+^, and IFN-γ^+^CD8^+^ cells, were significantly decreased (Figure 6D-E and Figure S5A). Consistent with reduced IFN-γ-producing T cells, serum IFN-γ levels were also significantly lower (Figure 6F-G). Moreover, expression of other effector molecules (TNF-α, CD107a, FasL, perforin, GZMB) was downregulated (Figure S5B). These results suggest that PD-L1 Fc therapy selectively targets pathogenic CD8^+^ T cell subsets in murine LN.

### PD-L1 Fc suppresses pathogenic CD8^+^ T cell activation *in vitro*

**Figure 7:**
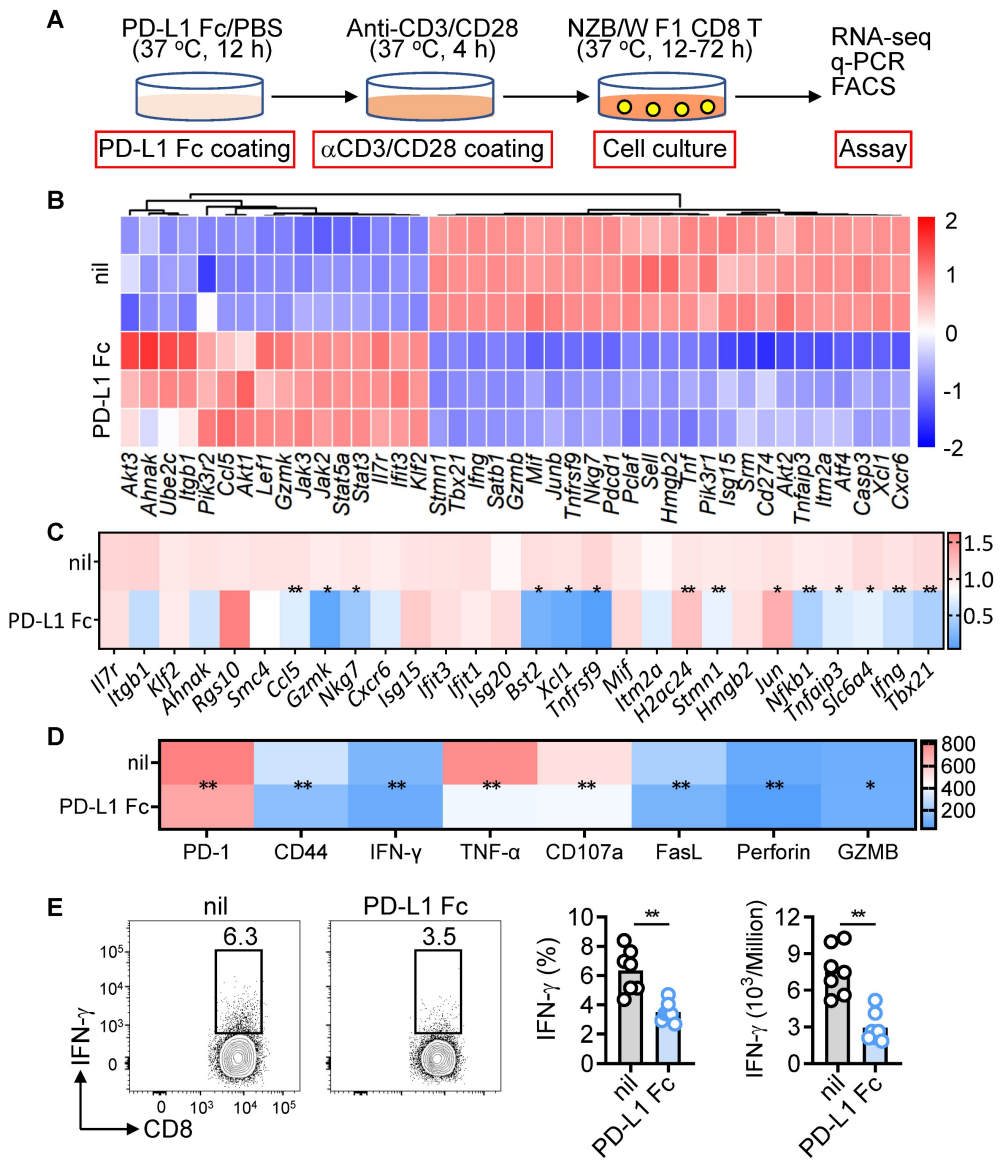
PD-L1 Fc suppresses CD8^+^ T cell activation and function *in vitro*. (A) Experimental design for CD8^+^ T cells from NZB/W F1 mice stimulated with anti-CD3/CD28 in the presence or absence of PD-L1 Fc. (B, C) Gene expression analysis after PD-L1 Fc treatment (12 h), including bulk RNA-seq (B) and q-PCR validation of selected genes (C). (D, E) FACS analysis of activation markers, cytokines, and cytotoxic molecules (MFI) (D), and IFN-γ ratio in CD8^+^ T cells treated with or without PD-L1 Fc (72 h) (E). Data are shown as individual points with mean ± SEM and analyzed by Mann-Whitney U-test (C-E). *p ≤ 0.05, **p ≤ 0.01.

Given the inhibitory effects of PD-L1 Fc *in vivo*, we evaluated its impact *in vitro*. CD8^+^ T cells from NZB/W F1 mice were activated with anti-CD3/CD28, followed by PD-L1 Fc treatment (Figure 7A). RNA-seq analysis revealed significant downregulation of genes related to activation, cytotoxicity, interferon signaling, migration, proliferation, and inflammatory pathways (Figure 7B). q-PCR and flow cytometry confirmed reduced transcripts and protein levels of effector molecules (Figure 7C-D). PD-L1 Fc also significantly decreased both the frequency and number of IFN-γ-producing CD8^+^ T cells, highlighting its potent inhibitory effect on IFN-γ production (Figure 7E). These findings demonstrate PD-L1 Fc suppresses the activation and cytotoxicity of pathogenic CD8^+^ T cells *in vitro* and *in vivo*.

### PD-1-Stat1-T-bet axis regulates IFN-γ production in CD8^+^ T cells

**Figure 8:**
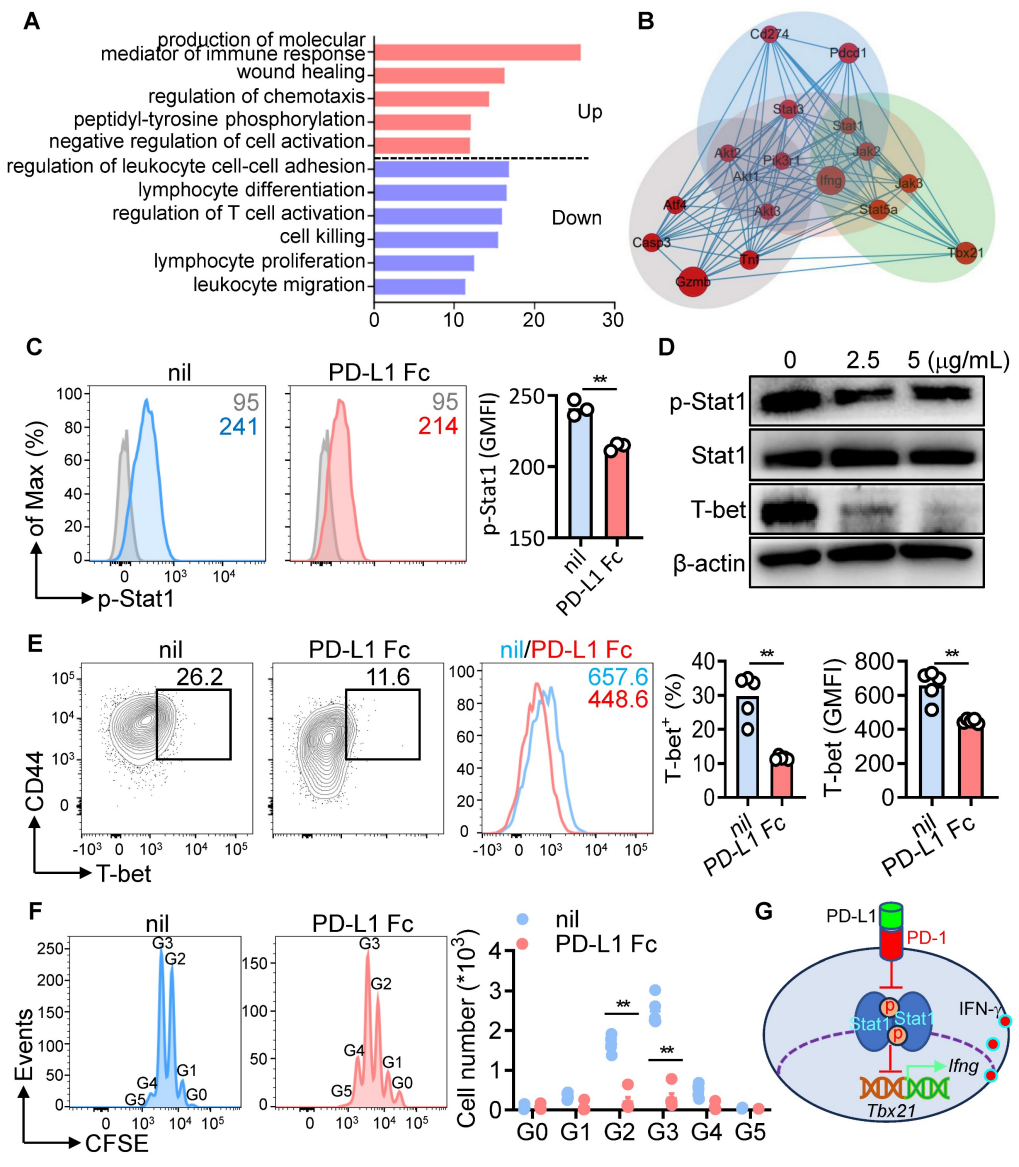
PD-1-Stat1-T-bet axis regulates IFN-γ production in CD8^+^ T cells. (A) GO analysis of differentially expressed genes in PD-L1 Fc-treated CD8^+^ T cells (from Figure 7B). (B) Schematic of the PD-1-Stat1-T-bet-IFN-γ pathway. (C) FACS analysis of phosphorylated Stat1 (p-Stat1) in CD8^+^ T cells treated with PD-L1 Fc. (D) Western blot of p-Stat1, total Stat1 (3 h), and T-bet (12 h) in PD-L1 Fc-treated CD8^+^ T cells. (E, F) FACS analysis of T-bet expression (E) and CD8^+^ T cell proliferation (F) after PD-L1 Fc treatment (72 h). (G) Schematic of PD-1/PD-L1 interaction in regulating IFN-γ secretion by CD8^+^ T cells. Data are shown as individual points with mean ± SEM and analyzed by Mann-Whitney U-test (C, E, F). *p ≤ 0.05, **p ≤ 0.01.

To uncover how PD-1 signaling regulates IFN-γ production, Gene Ontology (GO) analysis was performed on PD-L1 Fc-treated CD8^+^ T cells. Immune response mediators and negative regulation of cell activation were upregulated, whereas pathways linked to activation, cytotoxicity, and migration were downregulated (Figure 8A). Network analysis identified the Stat1-T-bet-IFN-γ pathway as a key target of PD-1-PD-L1 interaction (Figure 8B). CD8^+^ T cells from NZB/W F1 mice after anti-CD3/CD28 activation, PD-L1 Fc treatment significantly reduced phosphorylated Stat1 (p-Stat1), as confirmed by phospho-flow cytometry and Western blot in (Figure 8C-D). T-bet, a transcription factor driving IFN-γ expression 38, was also downregulated (Figure 8E), particularly in proliferating CD8^+^ T cells (Figure 8F). These findings suggest that PD-1 signaling inhibits p-Stat1, leading to reduced T-bet expression and decreased IFN-γ production (Figure 8G).

## Discussion

Our study underscores that PD-1, despite generally transmitting inhibitory signals, also functions as an activation marker in CD8⁺ T cells. We identified a hyperactivated subset of PD-1^+^CD8^+^ T cells in LN patients and NZB/W F1 mice, characterized by increased activation, cytokine production, and cytotoxicity. This aligns with PD-1 being an activation marker, with these cells not yet in a state of terminal exhaustion. Hence, this highlights the complex role of PD-1 signaling in CD8^+^ T cell function in autoimmune diseases.

Single-cell RNA sequencing revealed a diverse landscape of PD-1^+^CD8^+^ T cell subsets, including clusters with features of CD29, cytotoxicity, ISGs, proliferation, and Junb/NF-κB pathway activation. Notably, CD29 mediates immune cell migration and tissue adhesion 39, and CD29⁺CD8⁺ subsets have been associated with elevated serum IgG levels 40 and heightened cytotoxic capacity 31. Their expansion in the kidney of mice with advanced LN suggests a pathogenic role in tissue damage. Similar findings have been reported in patients with juvenile idiopathic arthritis (JIA), where PD-1^+^CD8^+^ T cells exhibited tissue-resident memory (Trm) profiles and clonal expansion, driving chronic inflammation 41. PD-L1 Fc treatment ameliorated nephritis by inhibiting these hyperactivated clusters, emphasizing their potential as therapeutic targets.

The variability in CD8^+^ T cell phenotypes across LN models emphasizes the impact of genetic background and disease context. In MRL/*lpr* mice, kidney-infiltrating CD8^+^ T cells appear metabolically exhausted 24, whereas in NZB/W F1 mice, PD-1^+^CD8^+^ T cells are hyperactivated. This discrepancy may stem from factors such as defective apoptosis and persistent autoantigen exposure in MRL/*lpr* mice 42, 43. Genetic variations in PD-1 signaling also influence disease outcomes, as evidenced in different PD-1-deficient mouse strains and *PDCD1* polymorphisms in LN patients 17, 19, 44, 45.

The elevated PD-1 expression on CD8⁺ T cells in SLE patients and murine model likely arises from continuous antigenic stimulation combined with a proinflammatory environment. Notably, kidney-infiltrating CD8⁺ T cells exhibit significantly higher PD-1 expression than circulating CD8⁺ T cells, suggesting that the kidney's inflammatory microenvironment and chronic autoantigen exposure promote PD-1 upregulation and enhance effector function. Sustained exposure to proinflammatory cytokines and autoantigens further elevates PD-1 expression, resulting in a PD-1^hi^ phenotype. These PD-1^hi^ cells are responsive to PD-L1 Fc treatment, which suppresses CD8⁺ T cell activity, thereby protecting the kidney and improving renal function.

Targeting PD-1 and CTLA-4, has shown promise in treating autoimmune diseases like RA 46, 47. Nevertheless, the mechanisms differ between CD4^+^ and CD8^+^ T cells. In cancer immunotherapy, PD-1 blockade predominantly enhances CD8^+^ T cell responses, whereas CTLA-4 inhibition primarily affects CD4^+^ T cells 48. Similarly, targeting CD4^+^ or CD8^+^ T cells with 5-azacytidine improves lupus pathology through distinct pathways in MRL/*lpr* mice 35. Our study corroborates these findings, showing that PD-L1 Fc selectively suppresses pathogenic CD8^+^ T cells, Th1 and Tfh cells while promoting Tregs in NZB/W F1 mice.

In conclusion, our findings reiterate that PD-1 is primarily an inhibitory checkpoint, and that the observed hyperactivation of PD-1^+^CD8^+^ T cells reflects a stage before terminal exhaustion, with PD-L1 ligation attenuating this activation. These results underscore the therapeutic potential of targeting PD-1 signaling to manage autoimmune diseases like LN.

## Supplementary Material

Supplementary figures and tables.

## Abbreviations

CD: cluster of differentiation
FasL: Fas ligand
gMFI: geometric mean fluorescence intensity
GO: gene ontology
GZMB: Granzyme B
H&E: hematoxylin and eosin
IFN-γ: interferon gamma
IL: interleukin
LN: lupus nephritis
PAS: Periodic Acid-Schiff
PBMC: peripheral blood mononuclear cells
PBS: phosphate-buffered saline
PD-1: programmed cell death-1
PD-L1: programmed death ligand 1
q-PCR: quantitative polymerase chain reaction
scRNA-seq: single-cell RNA sequencing
SLE: systemic lupus erythematosus
Stat1: signal transducer and activator of transcription 1
T-bet: T-box transcription factor
TCR, T cell receptor: UMAP, uniform manifold approximation and projection
W: week
