# Supplementary material for: PD-1 activation mitigates lupus nephritis by suppressing hyperactive and heterogeneous PD-1+CD8+ T cells
Source: Theranostics. 2025 Mar 31;15(11):5029–44. doi: 10.7150/thno.107418 (PMC12036892; doi:10.7150/thno.107418)
Supplement: Supplementary file 1 — Supplementary figures and tables. [file thnov15p5029s1.pdf]

Supplementary Materials

PD-1 activation mitigates lupus nephritis by suppressing hyperactive and heterogeneous PD-1$^+$CD8$^+$ T cells

Jun Deng[1,2,#,*], Junling Zhu[2,#], Xiaoyue Jiang[1,#], Chao Yao[1], Haifeng Chen[2], Yanjie Ding[3], Peng Niu[1], Qian Chen[4,*], Huihua Ding[1,*], Nan Shen[1,5,*]

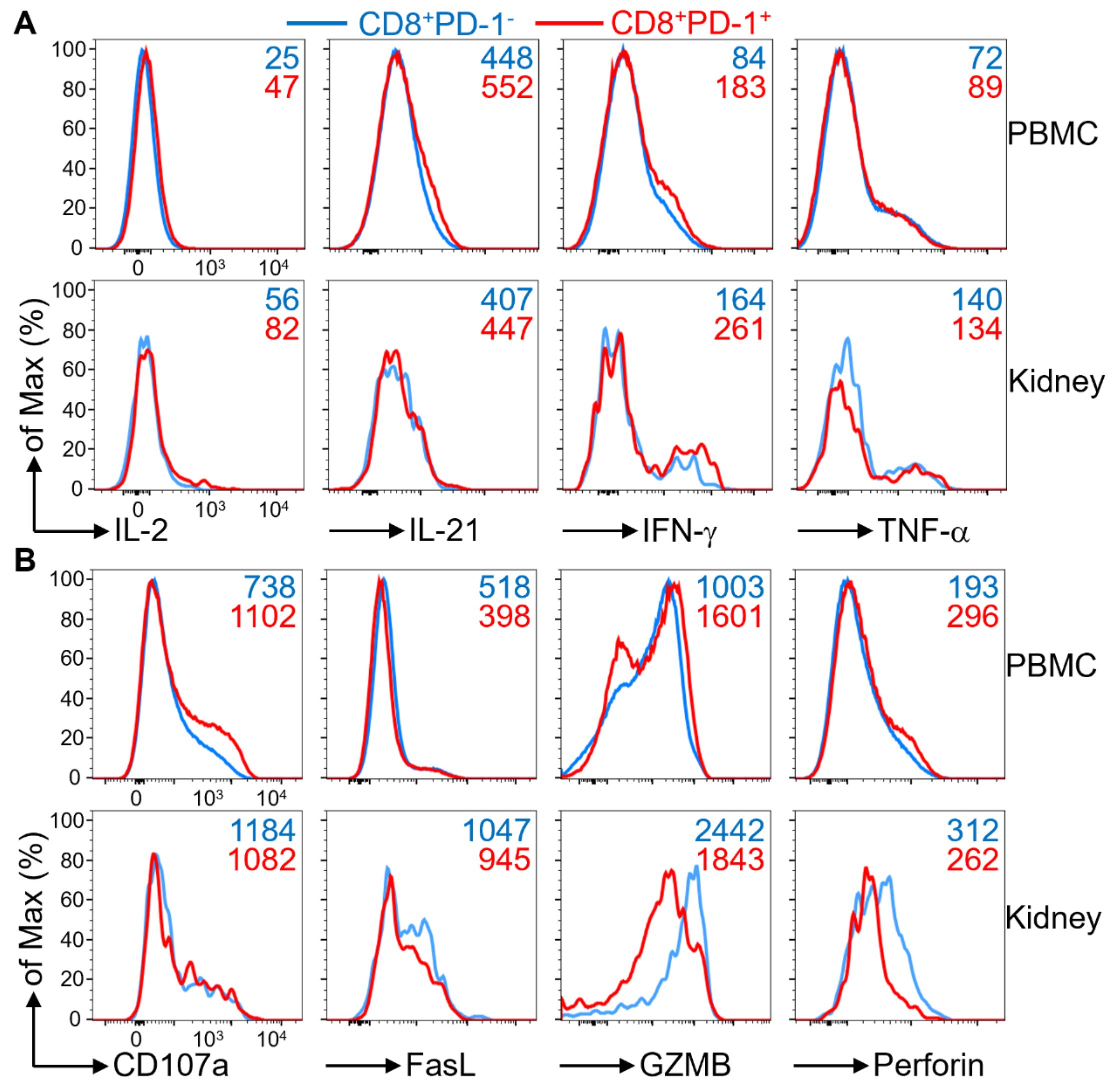

Figure S1. Cytokine production and expression of killing molecules by PD-1^{+} and PD-1^{-} CD8^{+} T cells from PBMCs and kidney MNCs. Flow cytometric histograms depicting the geometric mean fluorescence intensity (GMFI) values of cytokines (IL-2, IL-21, IFN-γ, TNF-α) (A), and killing molecules (CD107a, FasL, GZMB, perforin) (B), produced by PD-1^{+} and PD-1^{-} CD8^{+} T cells isolated from PBMCs ($n = 10$) and kidney MNCs ($n = 10$) of patients with LN.

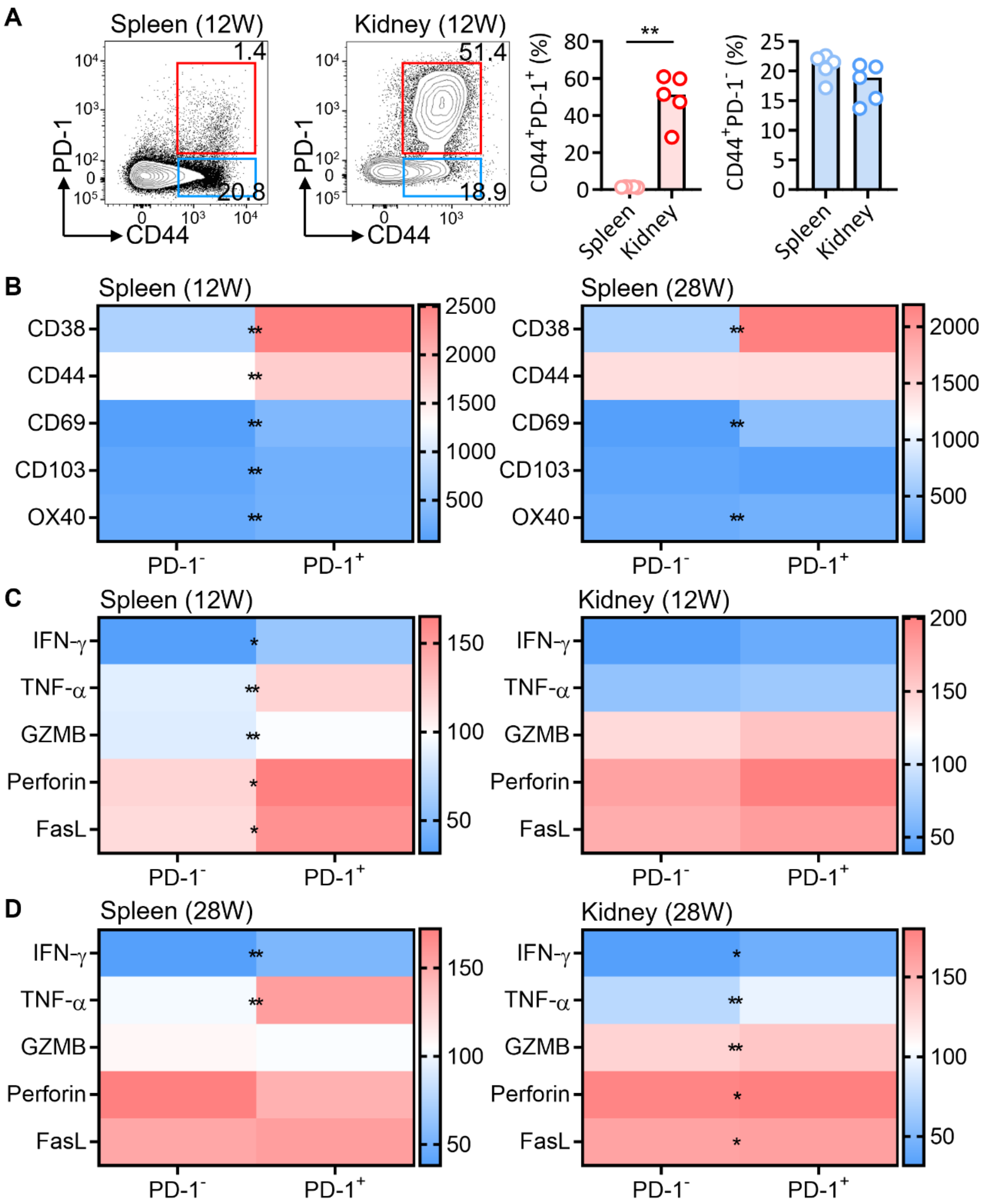

Figure S2. Elevated expression of activation markers on CD8$^+$PD-1$^+$ T cells compared to CD8$^+$PD-1$^-$ T cells in NZB/W F1 mice. (A-D) FACS plots and statistical analysis showing: (A) PD-1 expression on CD8$^+$CD44$^+$ cells from the spleen and kidney of 12-week-old (12W) female NZB/W F1 mice; (B) expression of activation markers (CD38, CD44, CD69, CD103, OX40) in the spleen at 12 and 28 weeks of age (12W and 28W); (C) cytokines

(IFN-γ, TNF-α) and cytotoxic molecules (GZMB, perforin, FasL) in PD-1^+ and PD-1^- cells from the spleen or kidney of 12W mice; and (D) data for 28W mice. Data are represented as individual values (dots, n = 5 per group) and mean (bars). Statistical analysis: Mann-Whitney U-test (A-D). *$p \leq 0.05$, **$p \leq 0.01$.

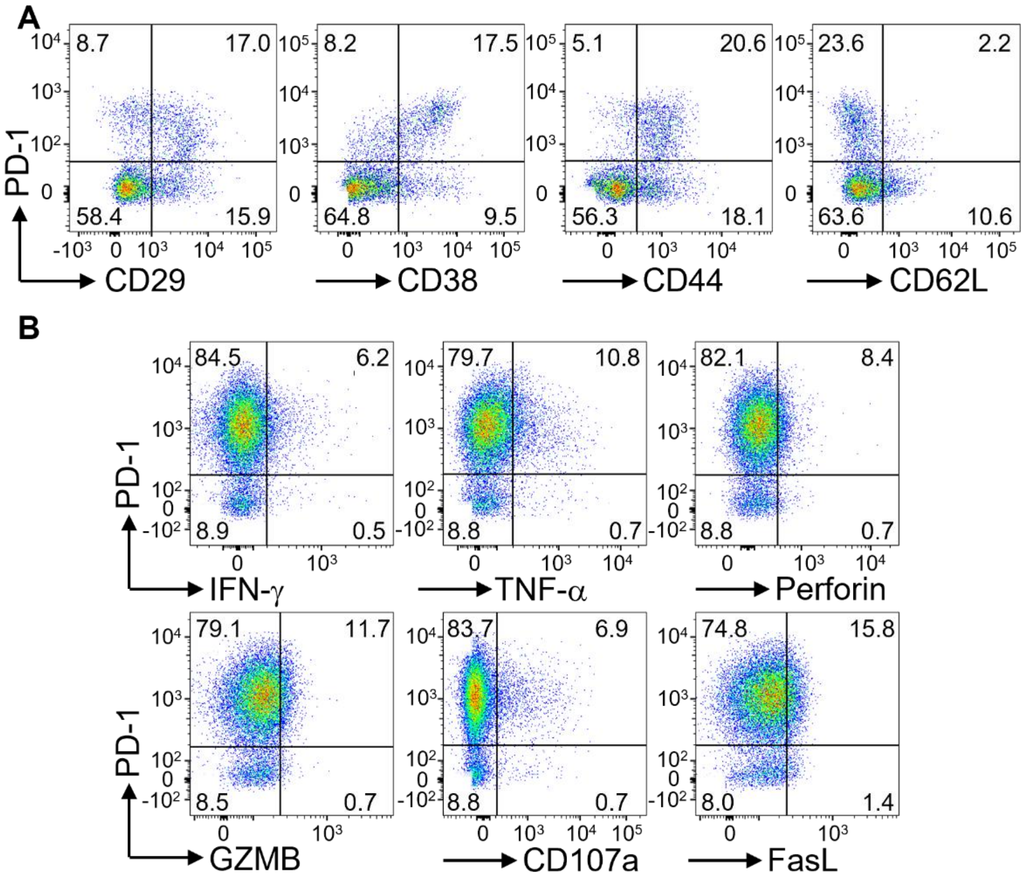

Figure S3. Co-expression of activation markers, cytokines, and cytotoxic molecules in PD-1^{+}CD8^{+} T cells in NZB/W F1 mice. (A, B) FACS plots showing the co-expression of PD-1 with activation markers, CD29, CD38, CD44, CD62L (A), cytokines (IFN-γ, TNF-α), and cytotoxic molecules (perforin, GZMB, CD107a, and FasL) (B) on CD8^{+} T cells in the spleen from NZB/W F1 mice at 28W.

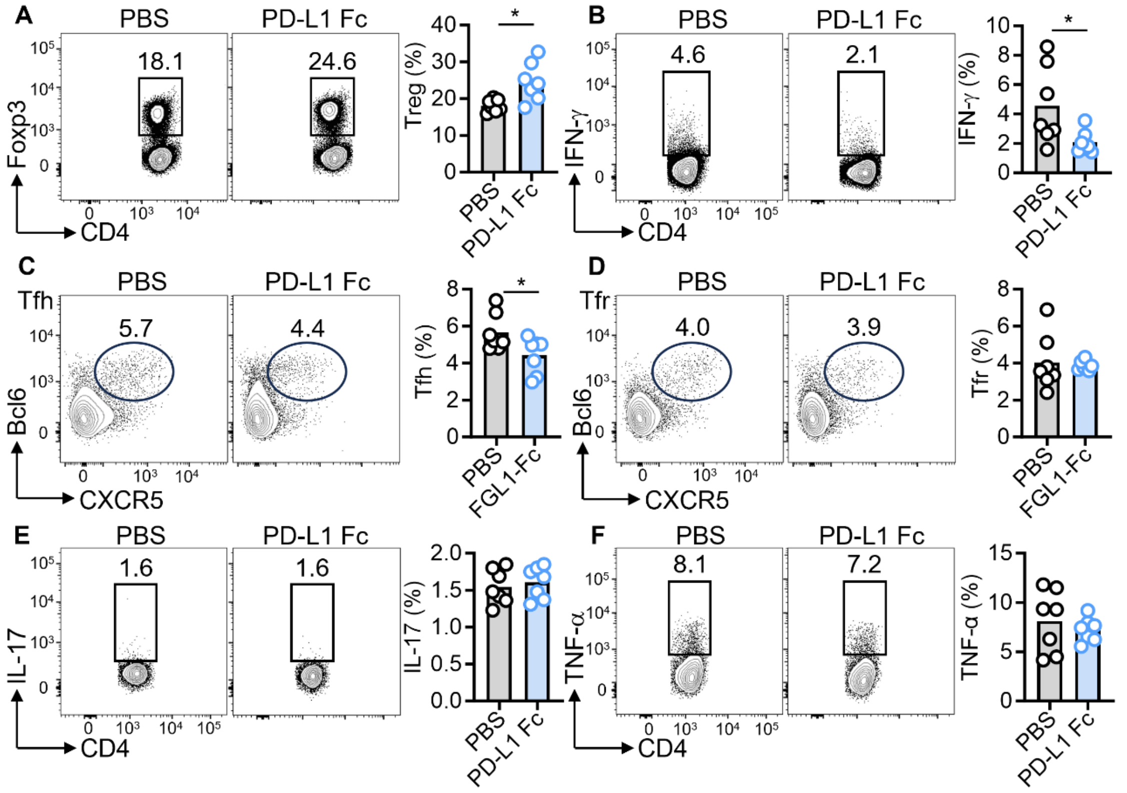

Figure S4. PD-L1 Fc reduces Th1 cells and promotes Treg cells in NZB/W F1 mice. (A, B) FACS plots and statistics showing the Treg cells (A), Th1 cells (B), Tfh cells (C), Tfr (D), Th17 cells (E) and TNF-α^{+}CD4^{+} T cells (F) in spleens from NZB/W F1 mice with PD-L1 Fc or IgG treatment (n = 7 per group). Data are shown for individuals (dots) and mean (bars) values. Statistical analysis: Mann-Whitney U-test (A-E). *$p \leq 0.05$.

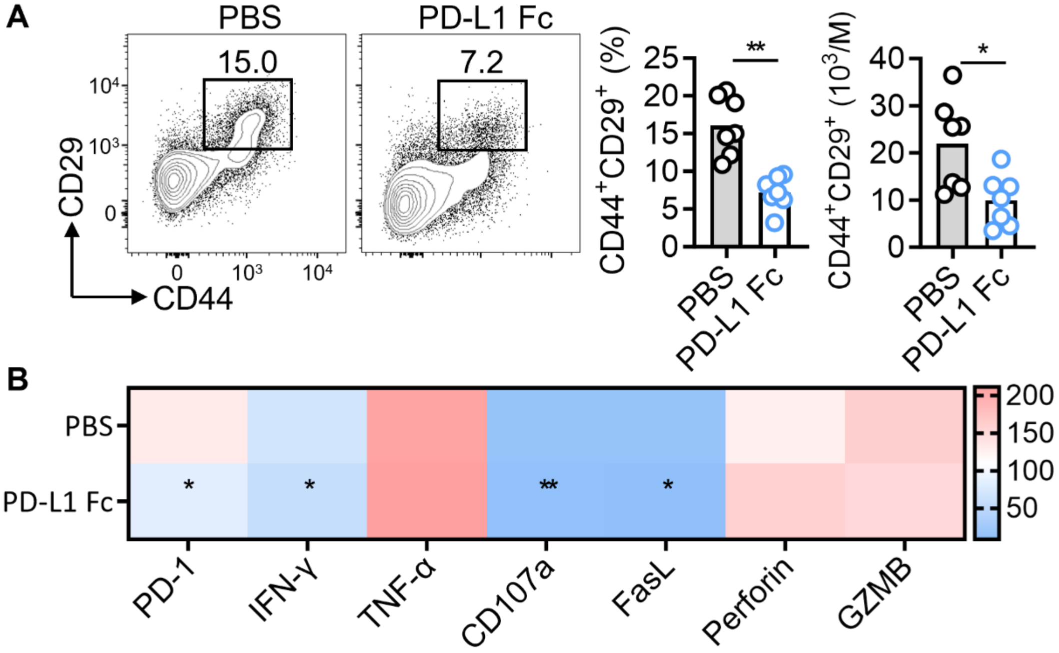

Figure S5. Reduction of CD44$^+$CD29$^+$ cells and effector molecule expression in CD8$^+$ T cells of NZB/W F1 mice after PD-L1 Fc treatment. (A, B) FACS plots and statistical analysis depicting: (A) frequencies and total numbers of CD44$^+$CD29$^+$CD8$^+$ T cells, along with CD29 and CD44 expression levels; (B) expression levels of PD-1, IFN-γ, TNF-α, CD107a, FasL, perforin, and GZMB in CD8$^+$ T cells from the spleen of mice treated with or without PD-L1 Fc (n = 7 per group). Data are represented as mean ± SEM. Statistical analysis: one-tailed Mann-Whitney U-test (A, B). *$p \leq 0.05$, **$p \leq 0.01$.

Table S1. Baseline characteristics of patients with SLE (n = 20)

Characteristics	Value
Age, year, median (range)	30.5 (19-45)
Female/Male	18/2
Weight, kg, median (range), n = 20	56 (43-75)
Height, cm, median (range), n = 20	160 (153-165)
Area, m^2, median (range), n = 20	1.6 (1.36-1.84)
Duration of SLE, year, median (range)	4 (0-12)
Prednisone dose, mg/day, median (range)	30 (10-60)
Use of concomitant agents (no. of patients)	
Hydroxychloroquine	5
Cyclophosphamide	1
Azathioprine	0
Cyclosporine	1
Methotrexate	0
Mycophenolate mofetil	2
Leflunomide	2
Thalidomide	0

Abbreviation: SLE, systemic lupus erythematosus.

Table S2. Clinical characteristics of patients with SLE (n = 20)

**Characteristics**	**value**
SELENA-SLEDAI, median (range)	12 (2-24)
Rash, n (%)	1 (5%)
Oral ulcers, n (%)	0 (0)
Serositis, n (%)	0 (0)
Raynaud's, n (%)	1 (5%)
Alopecia, n (%)	2 (10%)
Arthritis, n (%)	2 (10%)
Leukopenia, n (%)	2 (10%)
Thrombocytopenia, n (%)	1 (5%)
IgA, g/L, median (range)	2.41 (0.81-5.08)
IgG, g/L, median (range)	12.4 (3.59-33.1)
IgM, g/L, median (range)	0.98 (0.48-2.09)
C3, g/L, median (range)	0.529 (0.256-0.951)
C4, g/L, median (range)	0.0895 (0.029-0.187)
Anti-dsDNA, IU/mL, median (range)	96.97 (13.39-548.05)
Urine Protein, mg/24h, median (range)	2283.6 (48.6-10406.5)
Platelet, $\times 10^9$/L, median (range)	220 (98-315)
WBC, $\times 10^9$/L, median (range)	8.12 (3.06-18.56)
Lymphocyte, $\times 10^9$/L, median (range)	0.855 (0.27-2.67)
Monocyte, $\times 10^9$/L, median (range)	0.345 (0.02-0.89)
Neutrophil, $\times 10^9$/L, median (range)	6.9 (2.23-9.34)
Eosinophil, $\times 10^9$/L, median (range)	0.01 (0-0.13)

Abbreviation: C3/4, complement component 3/4; dsDNA, anti-double stranded DNA; g/L, gram/liter; Ig, immunoglobulin; IU/mL, international units per milliliter; mg/24h: milligram/24 hours; SELENA, safety of estrogens in lupus national assessment; SLEDAI, systemic lupus erythematosus disease activity index; WBC, white blood count.

Table S3. Primers for q-PCR assay

**Gene**	**Sense**	**Antisense**
Il7r	CACAGCCAGTTGGAAGTGGATG	GGCATTTCACTCGTAAAAGAGCC
Itgb1	CTCCAGAAGGTGGCTTTGATGC	GTGAAACCCAGCATCCGTGGAA
Klf2	CACCTAAAGGCGCATCTGCGTA	GTGACCTGTGTGCTTTCGGTAG
Rgs10	CGGAACCACAGCCTCCTTTCAA	TGCCATCCGTTCTGAAACCACG
Smc4	GTAGCGTACCAAAGAGACAGACG	CCTCTCATCACCTTGCTTCCAC
Ccl5	CCTGCTGCTTTGCCTACCTCTC	ACACACTTGGCGGTTCCTTCGA
Gzmk	CCATTCTCACGACTTCAGTCCG	TCACCTGGCATTTGGTCCCATC
Nkg7	CCACAGGTCCTCACTTCTCTGC	CAGCCAGGATACAGAAGCTCTG
Cxcr6	GGTTCTTCCTGCCATTGCTCAC	GCAGGAACACAGCCACTACAAG
Isg15	CATCCTGGTGAGGAACGAAAGG	CTCAGCCAGAACTGGTCTTCGT
Ifit3	GCTCAGGCTTACGTTGACAAGG	CTTTAGGCGTGTCCATCCTTCC
Ifit1	TACAGGCTGGAGTGTGCTGAGA	CTCCACTTTCAGAGCCTTCGCA
Isg20	GCCATTTGGTGAAGCCAGGCTA	AGCCTGTCTGTGGACGTGTCAT
Bst2	CAAACTCCTGCAACCTGACCGT	CTCCTGGTTCAGCTTCGTGACT
Xcl1	CAAGACCTATATCATCTGGGAGG	TGCCATCCACAGTCTTGATCGC
Tnfrsf9	CCAAGTACCTTCTCCAGCATAGG	GCGTTGTGGGTAGAGGAGCAAA
Mif	GAACCGCAACTACAGTAAGCTGC	ACGTTGGCAGCGTTCATGTCGT
Itm2a	GCAGGACTGATTGTTGGTGGAG	CTCCTCAGTCACAGGCAGAAAG
H2ac24	GCGACAACAAGAAGACGCGCAT	CTGGATGTTGGGCAGGACGCC
Stmn1	GAAGAAGGACCTTTCCCTGGAG	TGGCTTTCTGGAGCACCTCCTT
Hmgb2	GATGTGGTCTGAGCAATCTGCC	CCTGCTTCACTTTTGCCCTTGG
Jun	CAGTCCAGCAATGGGCACATCA	GGAAGCGTGTTCTGGCTATGCA
Nfkb1	GCTGCCAAAGAAGGACACGACA	GGCAGGCTATTGCTCATCACAG
Tnfaip3	AGCAAGTGCAGGAAAGCTGGCT	GCTTTCGCAGAGGCAGTAACAG
Slc6a4	GTTGATGCTGCGGCTCAGATCT	GAAGCTCGTCATGCAGTTCACC
Ifng	CAGCAACAGCAAGGCGAAAAAGG	TTTCCGCTTCCTGAGGCTGGAT
Tbx21	CCACCTGTTGTGGTCCAAGTTC	CCACAAACATCCTGTAATGGCTTG
Actb	GCGTGACATCAAAGAGAAGCT	ATGCCACAGGATTCCATACC
